# Overview and risk factors for postcraniotomy surgical site infection: A four-year experience

**DOI:** 10.1017/ash.2021.258

**Published:** 2022-01-31

**Authors:** Cristina Corsini Campioli, Douglas Challener, Isin Y. Comba, Aditya Shah, Walter R. Wilson, M. Rizwan Sohail, Jamie J. Van Gompel, John C. O’Horo

**Affiliations:** 1 Division of Infectious Diseases, Department of Medicine, Mayo Clinic, Rochester, Minnesota; 2 Department of Otolaryngology—Head and Neck Surgery and Neurologic Surgery, Mayo Clinic, Rochester, Minnesota; 3 Division of Pulmonary and Critical Care, Mayo Clinic, Rochester, Minnesota; 4 Section of Infectious Diseases, Baylor College of Medicine, Houston, Texas

## Abstract

**Objective:**

Despite evidence favoring perioperative antibiotic prophylaxis (ABP) use in patients undergoing craniotomy to reduce rates of surgical site infections (SSIs), standardized protocols are lacking. We describe demographic characteristics, risk factors, and ABP choice in patients with craniotomy complicated with SSI.

**Design:**

Retrospective case series from January 1, 2017, through December 31, 2020.

**Setting:**

Tertiary-care referral center.

**Patients:**

Adults who underwent craniotomy and were diagnosed with an SSI.

**Methods:**

Logistic regression to estimate odds ratios and 95% confidence intervals to identify factors associated with SSIs.

**Results:**

In total, 5,328 patients undergoing craniotomy were identified during the study period; 59 (1.1%) suffered an SSI. Compared with non-SSI cases, patients with SSI had a significantly higher frequency of emergency procedures: 13.5% versus 5.8% (*P* = .02; odds ratio [OR], 2.52; 95% confidene interval [CI], 1.10–5.06; *P* = .031). Patients with SSI had a higher rate of a dirty (5.1% vs 0.9%) and lower rate of clean-contaminated (3.3% vs 14.5%) wound class than those without infection (*P* = .002). Nearly all patients received ABP before craniotomy (98.3% in the SSI group vs 99.6% in the non-SSI group; *P* = .10). Combination of vancomycin and cefazolin as dual therapy was more prevalent in the group of patients without infection (n = 1,761, 34.1%) than those with SSI (n = 4, 6.8%) (*P* < .001), associated with decreased odds for SSI (OR, 0.17; 95% CI, 0.005–0.42; *P ≤* .001).

**Conclusions:**

SSI are frequently seen after an emergent neurosurgical procedure and a dirty wound classification. Combination of prophylactic cefazolin and vancomycin is associated with decreased risk for SSI.

Disruption of the mechanical barriers around the brain, such as open cranial fracture and neurosurgery, are well-known risk factors associated with infections.^1^ Common infectious complications after intracranial neurosurgical procedures include meningitis, subdural empyema, brain abscess,^2^ and occasionally surgical site infections (SSIs).^3^ Surveillance data suggest that the incidence of SSI is increasing^4^ because the number of neurosurgical procedures performed in the United States also continues to rise. The reported SSI rates following craniotomy are relatively variable. According to the National Healthcare Safety Network (NHSN) data in 2019, the national standardized infection ratio was 1.213, with a rate of 1.32%.^5^

Emergency, clean-contaminated or dirty surgery, operative time >4 hours, reoperation, and implanted materials are independent predictive factors for postoperative infections after craniotomy.^4,6–8^ On the contrary, clean neurosurgical procedures with no implementation of prosthetic devices carry a low risk of infection,^9^ and the risk of a prophylactic antibiotic side effect and adverse drug reaction could be unacceptable if the benefit is low. Nevertheless, the consequences of infections in neurosurgery represent a threat, requiring immediate medical and surgical intervention. Therefore, with the availability of newer antimicrobial agents and a previous case series from 1979, which showed the absence of infection in 1,732 major clean neurosurgical operative cases where antibiotic prophylaxis (ABP) was administered,^10^ many neurosurgeons routinely use prophylaxis in clean surgical procedures.

Nearly half of SSIs are preventable by implementing evidence-based strategies like parenteral ABP, glycemic control, perioperative normothermia, adequate volume replacement, and antiseptic prophylaxis at least the night before the operative day^11^. Although 2 meta-analyses^12,13^ and 8 randomized studies^14–21^ have concluded in favor of perioperative ABP for craniotomy in decreasing infection rates, there is a lack of protocols for this practice. Therefore, we have described the demographic characteristics, microbiology, variable ABP regimens, and risk factors associated with SSI in a contemporary cohort of patients who underwent a craniotomy at our institution.

## Methods

We retrospectively reviewed all adult (≥18 years of age) patients who underwent primary craniotomies and were diagnosed and not diagnosed with SSI at our institution between January 1, 2017, and December 31, 2020. Patients were identified using *International Classification of Disease, Tenth Revision* (ICD-10) codes for craniotomy and SSI.^22^ The medical records of those classified with SSI were manually revised. Patients’ electronic health records were reviewed, including demographic and clinical data, Charlson comorbidity index (CCI), American Society of Anesthesiologists classification of Physical Status (ASA) score, length of hospital stay (LOS), surgical diagnosis, emergency, wound contamination class, surgical procedure duration, and prophylactic antibiotic therapy used. All patients had consented to use their medical records for research purposes, and the study was approved by the Mayo Clinic Institutional Review Board.

A search was conducted using an advanced cohort explorer tool developed by Mayo Clinic using the term “craniotomy” and “surgical site infection.” According to the Centers for Disease Control (CDC) guidelines, we defined surgical site infections as those occurred near or at the incision site or deeper underlying tissue spaces and organs within 30 days of a surgical procedure, or up to 90 days for implanted prosthetics.^7,11^ We excluded cases where infection was suspected prior to the procedure. ASA status classification was defined as a system for assessing the fitness of a patient’s physiological status to help predict operative risk.^23^ Wound class was defined as the degree of contamination of a surgical wound at the operation time.^7^ Antibiotic prophylaxis was defined as an antibiotic regimen used before contamination by surgical incision has occurred and given with the intention of preventing infection.^7,24^ According to our institutional protocol, common antibiotics included cefazolin, given <1 hour prior to incision. Vancomycin started within 1–2 hours prior to incision. Clindamycin was given <1 hour prior to incision. Antibiotics were stopped 24 hours after the procedure. Microbiologic information that included infection culture results and 16S broad-range ribosomal RNA PCR and sequencing was collected. Types of pre-existing central nervous system (CSF) hardware included metal plates, ventriculoperitoneal shunts, leads, and electrodes.

### Statistical analysis

Descriptive information about patients who underwent craniotomy was reported as frequencies and proportions for categorical variables or median (interquartile range [IQR]) and mean (standard deviation [SD]) for continuous variables. Kruskal-Wallis rank-sum test and the Fisher exact test were used whenever appropriate. Statistical tests were 2-tailed, with *P* < .05 considered statistically significant. To identify factors associated with SSI, we used logistic regression to estimate odds ratios (ORs) and 95% confidence interval (CI). Variables included in the logistic regression model included age, sex, surgery duration, whether the surgery was an emergency surgery, the CCI, and the prophylactic antibacterial regimen. Other variables were not considered for inclusion in the model due to concerns of collinearity with the CCI (BMI, diabetes mellitus, ASA classification) and few numbers (wound classification, endoscopic approach). To fulfill the assumptions of logistic regression, continuous variables (age, CCI, and duration of surgery) were assessed for a linear relationship with the log odds of an SSI. Follow-up in the study period was complete. This model was purely for descriptive purposes. All observations were independent. Statistical analysis was performed using R, version 4.1.024.^25^

## Results

Demographics and surgical characteristics of patients who underwent craniotomy are summarized in Table 1. In total, 5,328 adult patients had craniotomy during the study period. Of these, 59 (1.1%) suffered an SSI. There were no statistically significant differences in the demographic characteristics between patients with and without SSI. The median patient age for those with SSI was 51 years (interquartile range [IQR], 36–65.5); the median patient age for those without an SSI was 56 years (IQR, 40–66) (*P* = .20). Most were males in both groups (57.6% vs 50.9%; *P* = .30). Compared with the cases without infection, patients with SSI had a higher body mass index (BMI) of 29.2 (IQR 25.7–35.4) versus 27.9 kg/m^2^ (IQR, 24–32.5; *P* = .07) and CCI score (1 ± 1.8 vs 0.7 ± 1.9; *P* = .07); however, the absolute rate of diabetes mellitus in the patients with SSI was lower than those with non-SSI (0% vs 5%; *P* = .10).

**Table 1.** Demographics and Surgical Characteristics of Patients Undergoing Craniotomy

Characteristic	No Surgical Site Infection (n = 5269)	Surgical Site Infection (n = 59)	*P* Value
Age, median y (IQR)	56 (40–66)	51 (36–65.5)	.20^a^
Male, no. (%)	2,685 (50.9)	34 (57.6)	.30^b^
BMI median kg/m^2^ (IQR)	27.9 (24.1–32.5)	29.2 (25.7–35.4)	.07^a^
CCI, mean (SD)	0.7 (1.9)	1 (1.8)	.07^a^
Diabetes mellitus, no. (%)	267 (5.1)	0 (0)	.10^b^
**ASA classification, no. (%)**			.8
I	66 (1.2)	0 (0)	
II	2,149 (40.8)	25 (42.4)	
III	2,759 (52.4)	30 (50.8)	
IV	236 (4.5)	4 (6.7)	
V	59 (1.1)	0 (0)	
**Wound classification, no. (%)**			**.002** ^c^
Clean	4,438 (84.2)	54 (91.5)	
Clean-contaminated	768 (14.5)	2 (3.3)	
Contaminated	12 (0.2)	0 (0)	
Dirty	51 (0.9)	3 (5.1)	
**Surgical diagnosis, no. (%)**			.40^c^
CSF shunt surgery	591 (11.2)	7 (11.8)	
Functional surgery	543 (10.3)	4 (6.7)	
Glial tumor	1,376 (26.1)	13 (22)	
Head surgery	450 (8.5)	5 (8.4)	
Meningioma	991 (18.8)	13 (22)	
Metastasis	276 (5.2)	5 (8.4)	
Neurinoma	271 (5.1)	0 (0)	
Subdural hematoma	331 (6.2)	6 (10.1)	
Vascular surgery	440 (8.3)	6 (10.1)	
Hardware implantation	126 (2.4)	2 (3.4)	
Emergency procedure, no. (%)	309 (5.8)	8 (13.5)	**.02** ^b^
Endoscopic approach, no. (%)	753 (14.3)	4 (6.7)	.10^b^
Duration of surgery, median h (IQR)	2.7 (1.6–4.3)	2.8 (1.6–4.4)	.70^a^
Hospital LOS, median d (IQR)	2 (2–4)	2 (2–3)	.10^a^

Note. ASA, American Society of Anesthesiologists; BMI, body mass index; CCI, Charlson comorbidity index; CNS, central nervous system; CSF, cerebrospinal fluid; IQR, interquartile range; LOS, length of stay;SD, standard deviation.

a Kruskal-Wallis rank-sum test.

b Fisher exact test for count data.

c Fisher exact test for count data with simulated *P* value.

The most common indications for craniotomy in the cases complicated with SSI included glial tumor (22%), meningioma (22%), CSF shunt surgery (11.8%), subdural hematoma (10%), and vascular surgery (10%); whereas for the cases without infection, indications encompassed glial tumor (26%), meningioma (18.8%), and CSF shunt surgery (11%) (*P* = .40). Hardware implantation cases were uncommon (n = 128, 2.4%), and 2 cases (1.6%) were complicated with SSIs.

Compared to the patients without infection, the frequency of emergency procedures was significantly higher in cases with SSI (13.5% vs 5.8%; *P* = .02), with a median duration of 2.8 (IQR, 1.6–4.4) versus 2.7 hours (IQR, 1.6–4.3), respectively (*P* = .70). The endoscopic approach was more common in cases without SSI (14.3% vs 6.7%; *P* = .10) than those with SSI.

There was no significant difference in the ASA classification between comparison groups, class III being the most frequent (50.8% vs 52.4%; *P* = .80). On the contrary, patients with SSI had a significantly higher rate of a dirty wound class [n = 3 (5.1%) vs n = 51 (0.9%)] and lower rate of clean-contaminated wound class [n = 2 [3.3%] vs n = 768 (14.5%)] compared to those without infection (*P* = .002). The mean time from craniotomy to the SSI occurrence was 26 days (SD, 16.4). Hospital LOS was similar in both groups, with a median of 2 days (*P* = .10).

### Antibiotic prophylaxis

Perioperative antibiotic selection is summarized in Table 2. Most patients received ABP before craniotomy (58 [98.3%] in SSI group vs 5251 [99.6%] in non-SSI group; *P* = .10). The most common antibiotic used was cefazolin (47 [81%] vs 2659 [51.4%]), followed by vancomycin (6 [10.3%] vs 578 [11.1%]), respectively. The combination of vancomycin and cefazolin as dual therapy was most common in the group of patients without infection (n = 1,761, 34.1%) than those with SSI (n = 4, 6.8%) (*P* < .001).

**Table 2.** Perioperative Prophylactic Antibiotic Selection in Patients With Craniotomy

Antibiotic Treatment	No Surgical Site Infection (n = 5,269), No. (%)	Surgical Site Infection (n = 59), No. (%)	*P* Value
Perioperative antibiotic prophylaxis	5,251 (99.6)	58 (98.3)	.10^a^
**Perioperative antibiotic regimen**			**<.001** ^b^
Cefazolin	2,659 (51.4)	47 (81)	
Vancomycin	578 (11.1)	6 (10.3)	
Clindamycin	148 (2.8)	1 (1.7)	
Vancomycin + cefazolin	1,761 (34.1)	4 (6.8)	
Vancomycin + cefepime	23 (0.4)	0 (0)	

Note. SD, standard deviation.

a Fisher exact test for count data.

b Fisher exact test for count data with simulated *P* value.

### Risk for surgical site infections

Logistic regression predicting subsequent SSI is summarized in Table 3. Six independent risk factors were included: age, male sex, CCI, surgical duration, emergency procedure, and ABP regimen. An emergency procedure was the strongest risk factor associated with postoperative infection in the univariate (unadjusted OR, 2.52; 95% CI, 1.10 to 5.06; *P* = .031); however, the significance disappeared in multivariate model (unadjusted OR, 1.97; 95% CI, 0.73–4.53; *P* = .14). Additionally, no other risk factors increased the odds of SSIs in multivariate analysis. The use of vancomycin and cefazolin as a dual therapy was associated with decreased odds for SSI (adjusted OR, 0.17; 95% CI, 0.005–0.42; *P* ≤ .001). Although males, prolonged surgery, and those with higher CCI score made up most SSI cases, those were not statistically significant risk factors.

**Table 3.** Logistic regression predicting subsequent surgical site infections (univariate and multivariate analysis)

Characteristic	Univariate (Unadjusted)			Multivariate (Adjusted)		
	OR	95% CI	*P* Value	OR	95% CI	*P* Value
Age	0.99	0.98–1.00	.19	0.99	0.97–1.00	.09
Sex, male	1.31	0.78–2.22	.31	1.27	0.71–2.32	.40
Duration of surgery, median h (IQR)	1.01	0.90–1.12	.84	1.04	0.91–1.17	.50
Emergency procedure	2.52	1.10–5.06	**.031**	1.97	0.73–4.53	.14
Charlson comorbidity index	1.05	0.91–1.17	.50	1.05	0.91–1.18	.40
**Perioperative antibiotic regimen**			**<.001**			
Cefazolin	1	1 (reference)		1	1 (reference)	
Vancomycin	0.59			0.50		.20
Clindamycin	0.38	0.22–1.28		0.43	0.15–1.24	.40
Vancomycin + Cefazolin	0.13	0.02–1.76		0.17	0.02–1.99	**<.001**
Vancomycin + Cefepime	0.00	0.04–0.32		0.00	0.005–0.42	>.90

Note. OR, odds ratio; CI, confidence interval; IQR, interquartile range.

### Microbiology

Among the patients with SSI, 50 (84.7%) had a positive intraoperative culture result. The most common pathogens identified were *Staphylococcus aureus* (n = 14, 28%), including 11 methicillin-susceptible *Staphylococcus aureus* (MSSA) isolates (22%), and 3 methicillin-resistant *Staphylococcus aureus* (MRSA) isolates (6%), followed by 8 coagulase-negative staphylococci isolates (CoNS, 16%), 8 *Cutibacterium acnes* isolates (16%), 5 *Klebsiella* spp (10%), and 4 *Pseudomonas aeruginosa* (8%). Also, 19 cultured specimens (38%) had >2 bacterium isolated: CoNS (42%), *Cutibacterium acnes* (21%), *Staphylococcus aureus* (11%), and *Klebsiella* spp (11%). In total, 19 intraoperative pathogens identified (38%), including CoNS, *Klebsiella* spp, and *Cutibacterium acnes*, were identified exclusively using PCR and sequencing.

Among the 2 patients with hardware implantation, MSSA was identified in both cases. Of 59 patients, 4 (7%) had a secondary bloodstream infection.

## Discussion

The current study is one of the largest and contemporary cohorts describing risk factors, role, and choice of antibiotic therapy in preventing SSI in patients undergoing craniotomy.

### Demographics and risk factors

In our study, almost all patients received ABP (99.6%), and the rate of SSI was 1.1%, which is lower than the national average reported by the NHSN^5^ and the ranges described in the control groups of the randomized studies included in the meta-analyses by Fang et al.^13^ Purported risk factors for developing SSI include male sex, postoperative CSF leakage and drainage, duration of operation, number of early reoperations (>1), nontraumatic surgical diagnosis, and ASA score (>2). On the contrary, no relationship is usually detected between SSI and age, baseline comorbidities, emergency procedures, type of ABP, intracranial pressure monitors, or steroid use.^4,12,13^ The findings of our study revealed neither demographics nor reported comorbidities to be risk factors for SSIs. Diabetes mellitus has been associated with the reduced response of neutrophil function, T cells, and disorders of humoral immunity.^26^ The greater frequency of infections in diabetic patients is caused by the hyperglycemic environment that favors immune dysfunction. As seen in our study, despite the fact that diabetes mellitus has not been significantly seen in patient with SSI after undergoing craniotomy,^4^ the CDC recommends implementing perioperative glycemic control and using blood glucose target levels <200 mg/dL (category IA) to prevent SSIs in general.^7^ Similarly, it has been recognized that the adipose tissue participates actively in immunity, releasing various proinflammatory, anti-inflammatory factors, cytokines, and chemokines.^27^ In our cohort, patients with SSIs had higher BMIs than those without infection. However, this difference was not statistically significant. Thus, the lack of association between diabetes mellitus and obesity with SSIs in patients with craniotomies requires large prospective studies to further define the burden of infectious morbidity conferred by those factors.

### Surgical characteristics

Among the surgical diagnosis in our study, meningioma, subdural hematoma, and brain metastasis surgery appeared to be more frequently at risk than other reasons for craniotomy, which is like previous studies.^14,28^ However, 1 case-control study^29^ indicated a negative correlation with SSIs. The immunosuppression in patients with brain metastasis and the closure difficulties in subdural hematoma and meningioma surgeries may explain why nontraumatic surgeries lead to SSI, yet the exact mechanism is still not fully understood. Conversely, wound classification and emergency surgery are predisposing factors for SSI.^4^ However, all patients with emergency surgery received ABP in our study, and it was still associated with an increased risk for SSIs in the univariate analysis (OR, 2.52; 95% CI, 1.10–5.06). Notably, the use of prespecified criteria for diagnosing wound infection should lessen the bias resulting from the surgeon’s use of personal criteria.

Duration of surgery is a major factor in the National Nosocomial Infections Surveillance system report,^30^ reflecting either surgeon experience, surgical difficulties, or intraoperative complications. In our SSI cohort, the duration of surgery was somewhat longer than those without infection, but it was not a significant independent risk factor (OR, 1.01; 95% CI, 0.90–1.12). Our institution is a referral center, overall receiving more complex surgical cases, which may explain this observation.

Literature describing the infection rate following an endoscopic approach in neurosurgery is limited, especially restricted to SSIs. Kassam et al^31^ reported a rate of infectious complications of 1.9% in 800 patients undergoing endoscopic endonasal skull-base surgery. In our cohort, despite the small number of individuals having an endoscopic approach (14.2%), only 4 patients (0.53%) had an SSI, suggesting a virtual benefit with these surgical techniques. However, this finding was not significant and might be confounded by different patient characteristics and surgical circumstances.

### Microbiologic etiology of surgical site infections

Most reports agree that the most common organisms causing SSIs are MRSA, CoNS, *Pseudomonas aeruginosa*, and *Enterococcus* spp.^32,33^ However, Wang et al^34^ retrospectively analyzed >900 head and facial plastic surgery SSI cases.^34^ The most causative organisms in their study were *Pseudomonas aeruginosa* and *Klebsiella pneumoniae*, suggesting pathogen-specific prevalence based on the type of procedure. In our cohort, the most common offending organism was *Staphylococcus aureus*, followed by CoNS, and *Cutibacterium acnes*, which is concordant with previous reports.^35,36^ As seen in our study, commensal skin flora microorganisms account for many responsible bacteria in infected patients. An interesting finding in this study was that anaerobes, *Acinetobacter* spp, or *Candida* spp were not causes of SSI, differing from the findings of previous reports.^6,37,38^

### Antibiotic prophylactic therapy to prevent surgical site infections

To target and effectively prevent the infection caused by the most common microorganisms, the development of appropriate strategies, including ABP and infection prevention practices, are essential to improve the morbidity and mortality associated with SSI.^11^ The causative pathogens associated with SSIs in US hospitals have changed over the past 2 decades, including an increased proportion of MRSA^39^; hence, appropriate ABP effectiveness should be evaluated periodically based on the local prevalence. The American Society of Health-System Pharmacists therapeutic guidelines recommends using cefazolin, vancomycin, or clindamycin in neurosurgery.^40^ For procedures in which pathogens other than staphylococci and streptococci are likely, an additional agent with activity against those pathogens could be considered; however, the strength of recommendation varies from evidence from large, well-conducted, randomized, controlled clinical trials or a meta-analysis to expert opinion or data extrapolated from evidence for general principles and other procedures; leaving the ultimate antibiotic selection to the practitioner’s idiosyncrasy.

In our study, cefazolin was the most common antibiotic of choice for prophylaxis (52%), followed by the combination of cefazolin and vancomycin (34%). The combination prophylaxis was associated with decreased risk of SSIs (OR, 0.17; 95% CI, 0.005–0.42). *Staphylococcus aureus* and CoNS are 77% and 40% susceptible to oxacillin in our institution, respectively, potentially accounting for the decreased infection rate when vancomycin is added. Reported risk for devastating complications associated with infections in neurosurgery can be up to 17%,^41^ which may explain the practice variation in ABP selection. As this study was observational, we cannot draw any conclusion regarding the comparative efficacy of these regimens. Selection biases may have led to the broader antibiotic use. Prospective study protocols are indicated to better differentiate where alternative prophylaxis regimens may be appropriate.

This study had several limitations. The study’s retrospective nature with a case determination based on a claim data set is a potential limitation that over- or underestimate the incidence of SSI. Individual patient management was based on the discretion of attending physicians. Relatively few events limited the number of risk factors that could be statistically assessed. Because PCR is a highly sensitive technique, any sample contamination can produce misleading results. We did not examine individual bacterial isolates from the SSIs to assess the relationship between antimicrobial susceptibility and individual prophylactic regimen and antimicrobial side effects. Moreover, as a tertiary-care institution, our center has neurosurgery services readily available that may expedite management and could influence patient outcomes.

In conclusion, in our retrospective review, the combination of prophylactic cefazolin and vancomycin is associated with decreased risk for SSI. National guidelines and a prospective study evaluating the benefits and outcomes of monotherapy versus dual antimicrobial prophylaxis to prevent SSI in craniotomy is warranted.
